# An explainable-by-design end-to-end AI framework based on prototypical part learning for lesion detection and classification in Digital Breast Tomosynthesis images

**DOI:** 10.1016/j.csbj.2025.06.008

**Published:** 2025-06-10

**Authors:** Andrea Berti, Camilla Scapicchio, Chiara Iacconi, Charlotte Marguerite Lucille Trombadori, Maria Evelina Fantacci, Alessandra Retico, Sara Colantonio

**Affiliations:** a Institute of Information Science and Technologies (ISTI) - National Research Council of Italy (CNR), Via Giuseppe Moruzzi, 1, Pisa, 56127, Italy; b National Institute for Nuclear Physics (INFN) section of Pisa, Largo Bruno Pontecorvo, 3/Edificio C, Pisa, 56127, Italy; c Department of Information Engineering, University of Pisa, Via Girolamo Caruso, 16, Pisa, 56122, Italy; d Department of Physics, University of Pisa, Largo Bruno Pontecorvo, 3, Pisa, 56127, Italy; e Department of Radiology, Breast Imaging UOSD, Piazza Monzoni 1, Carrara, 54033, Italy; f Department of Diagnostic Imaging, Oncological Radiotherapy and Haematology, Fondazione Policlinico Universitario A. Gemelli IRCCS, Largo Agostino Gemelli, 8, Rome, 00168, Italy

**Keywords:** Ante-hoc explainability, Deep learning, ProtoPNet, XAI, DBT, Lesion detection, Lesion classification

## Abstract

**Background and Objective:**

Breast cancer is the most common cancer among women worldwide, making early detection through breast screening crucial for improving patient outcomes. Digital Breast Tomosynthesis (DBT) is an advanced radiographic technique that enhances clarity over traditional mammography by compiling multiple X-ray images into a 3D reconstruction, thereby improving cancer detection rates. However, the large data volume of DBT poses a challenge for timely analysis. This study aims to introduce a transparent AI system that not only provides a prediction but also an explanation of that prediction, expediting the analysis of DBT scans while ensuring interpretability.

**Methods:**

The study employs a two-stage deep learning process. The first stage uses state-of-the-art Neural Network (NN) models, specifically YOLOv5 and YOLOv8, to detect lesions within the scans. An ensemble method is also explored to enhance detection capabilities. The second stage involves classifying the identified lesions using ProtoPNet, an inherently transparent NN that leverages prototypical part learning to distinguish between benign and cancerous lesions. The system facilitates clear interpretability in decision-making, which is crucial for medical diagnostics.

**Results:**

The performance of the AI system demonstrates competitive metric results for both detection and classification tasks (a recall of 0.76 and an accuracy of 0.70, respectively). The evaluation metrics, together with the validation by expert radiologists through clinical feedback, highlight the potential of the system for future clinical relevance. Despite challenges such as dataset limitations and the need for more accurate ground truth annotations, which limit the final values of the metrics, the approach shows significant advancement in applying AI to DBT scans.

**Conclusions:**

This study contributes to the growing field of AI in breast cancer screening by emphasizing the need for systems that are not only accurate but also transparent and interpretable. The proposed AI system marks a significant step forward in the timely and accurate analysis of DBT scans, with potential implications for improving early breast cancer detection and patient outcomes.

## Highlights

• Digital Breast Tomosynthesis (DBT) powerful tool for detection of breast cancer.
• Development of transparent AI system with YOLO for lesion detection in DBT scans.
• ProtoPNet used for explainable classification of benign vs. cancerous lesions.
• Development of end-to-end framework for detection and classification of breast cancer.
• Clinical feedback by radiologists, showing clinical potential.

## Graphical abstract

**Figure:**
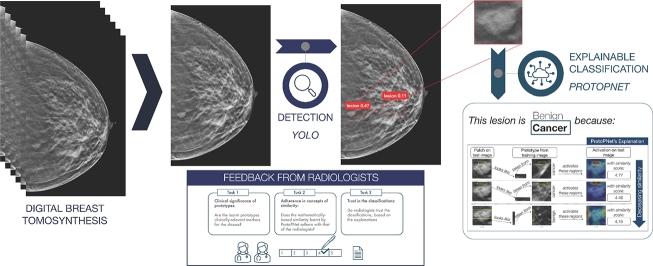

## 1 Introduction

Breast cancer is the leading cause of cancer-related deaths among women worldwide [28], [32], [11], [29], [36]. Early diagnosis is crucial to improving outcomes, making breast screening essential as the most dependable method for timely detecting breast cancer [31].

Digital Breast Tomosynthesis (DBT) enhances traditional mammography by capturing multiple X-ray images from different angles [8], allowing clearer visualization of breast tissue and improved cancer detection, especially in dense areas. However, the volume of images from DBT makes manual review time-consuming.

In this regard, Deep Learning (DL), which has achieved unparalleled efficacy in diverse domains, ranging from identifying marine objects [34] to powering self-driving vehicles [9], offers a way to streamline this process, thus reducing analysis time. In the panorama of object detection methods, recent versions of YOLO (You Only Look Once) models excel in areas such as detecting smaller objects and adapting to various datasets, and require a reduced computational cost [10]. Despite the success of DL in many domains, healthcare adoption remains cautious due to the ‘black-box’ nature of these models and their inherent intricacies, which could inadvertently introduce errors and biases, potentially resulting in disastrous consequences.

**Fig. 1:**
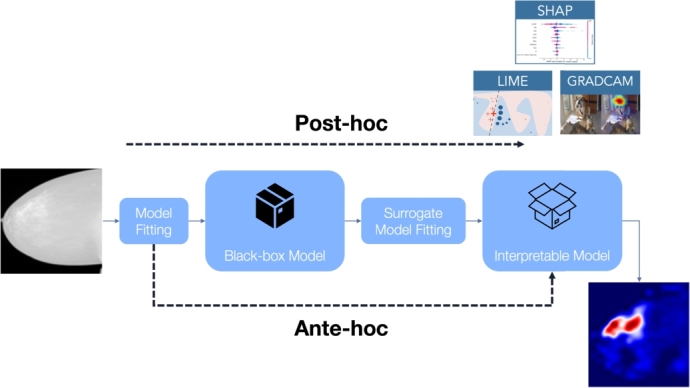
Post-hoc vs. ante-hoc explainability: post-hoc methods (such as Lime, SHAP, and Grad-cam) try to explain an already existing black-box model; on the other hand, ante-hoc explainability tries to build inherently-explainable models.

In recent times, eXplainable AI (XAI) addresses this challenge, aiming to make model decisions transparent. XAI approaches fall into two main categories [23], [1], [19] (see Fig. 1). The first, known as *post-hoc explainability*, focuses on interpreting the workings of a black-box AI model after its training and evaluation phase. Techniques within this category, such as LIME [22], SHAP [17], and Grad-cam [24], try to approximate the behavior of a black-box model by extracting relationships between the input features and the output predictions, and presenting them in a human-understandable way. However, the relationships found may not be the same as those exploited by the model. The latter is called *ante-hoc explainability* or *explainability by-design*. It proposes the development and use of an inherently interpretable model, creating a Neural Network (NN) that is explainable by-design, i.e., a model providing predictions, together with the rationale behind them. An example of an explainable-by-design NN is ProtoPNet [7], which introduces prototypical part learning, i.e., it classifies images based on learned visual prototypes. This means that in this approach, an image is classified by finding similarities between patches of previously learned cases that are representative of different classes, i.e., the prototypes, and patches of the query image itself.

**Fig. 2:**
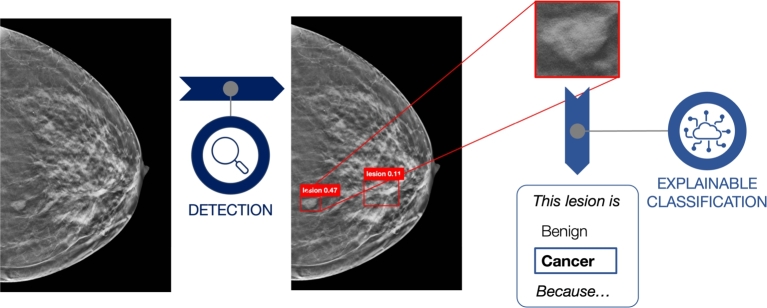
Visual representation of our proposed two-stage framework. Given a slice from a DBT scan, the detection module identifies the lesions. Subsequently, these lesions are cropped and fed into ProtoPNet, which performs *explainable classification* by indicating *why* a lesion is classified as benign or cancerous, based on its resemblance to learned prototypical patterns.

In this work, we propose a transparent two-stage AI framework that can locate and classify lesions on DBT images, while also explaining the rationale behind that classification. First, we use YOLOv5 and YOLOv8 (individually and as an ensemble) to detect lesions in 2D slices of DBT scans. Detected regions are then classified into benign and cancer lesions by ProtoPNet, which provides interpretable outputs based on prototypical similarities, therefore obtaining a classification of the form *“This lesion is of class Cancer, because this patch of the image resembles this prototype I learned during training”*. A visual representation of our two-stage framework is shown in Fig. 2.

To ensure clinical relevance and transparency, we involved two expert radiologists to assess ground truth annotation accuracy, evaluate the clinical relevance of false positives, and validate the interpretability of the outputs from ProtoPNet. This feedback ensures our AI system meets the high standards required for medical image analysis and maintains transparency in its decision-making processes.

Our contributions are threefold: (1) an end-to-end, interpretable framework for lesion detection in DBT; (2) application of ProtoPNet to a complex medical task; and (3) pioneering use of AI in the relatively new domain of DBT imaging.

### 1.1 Related work

The Breast Cancer Screening (BCS)-DBT dataset, which we employed in our study, was proposed in [5] as a comprehensive and publicly accessible dataset of DBT images. The dataset was used, in [16], to present the DBTex challenge, a multi-institutional international competition aimed at developing AI algorithms to detect lesions in DBT images. The challenge was divided into two phases, the first one providing just a subset of the dataset. A total of eight teams participated in the challenge. The results showed that the three best-performing teams, i.e., the NYU B-Team achieving the highest mean sensitivity for biopsied lesions at 0.957, followed by ZeDuS at 0.926, and, lastly, the VICOROB at 0.886, all achieved their scores by utilizing additional proprietary datasets. The other teams, who solely exploited the BCS-DBT dataset, had a mean sensitivity ranging from 0.814 and 0.390.

Beyond the challenge, other research has focused on lesion detection in DBT images. For instance, [13] utilized the BCS-DBT dataset and proposed using false positives from non-biopsied benign lesions as a form of data augmentation, training YOLOv5 to achieve a sensitivity of 0.80 with two false positives per image. Meanwhile, [12] employed, on a private dataset, a faster region-based convolutional NN (faster-RCNN) approach, achieving an auROC of 0.96 and outperforming DCNN-based methods in mass detection.

In terms of classification, few studies have tackled the more trivial classification of cancerous masses from normal cases using DBT images. [2] applied a self-attention graph CNN model, achieving an accuracy of 0.84 and an auROC of 0.87, while [30] used a trainable summarization module followed by a CNN, reaching an auROC of 0.73. Both of them add private datasets to the BCS-DBT one to improve performance.

Concerning the studies that tackle the same classification task we explore in our paper, i.e., lesion classification into benign and cancerous, to the best of our knowledge, at the time of our study, only one other work has investigated this, albeit with a different and proprietary dataset. The authors utilize, in [37], a two-dimensional CNN, pre-trained on ImageNet, as a feature extractor for each slice. Through a pooling process, they then generate a final classification for the whole 3D image. In their work, they achieve an auROC of 0.85.

It is crucial to note that direct quantitative comparisons of our results on DBT with studies employing 2D mammography are inherently challenging due to the significant differences in data acquisition (pseudo-3D volumetric data vs. 2D projections) and the resulting impact on tissue superposition and lesion visibility. Therefore, while we contextualize our work by discussing existing research on DBT and the application of explainable-by-design models to various medical imaging tasks, a direct numerical comparison across different imaging modalities would be misleading.

Regarding the use of an explainable by-design classifier, ProtoPNet has been applied to medical images only in a few works. Among those, it has been applied to the categorization of Alzheimer Disease from MRI images in [18], to COVID-19 detection from X-ray scans in [27], [25], and computed tomography in [26]. In [6], we explored the use of ProtoPNet in breast masses classification from mammogram images, demonstrating a recall of 0.769 and an AUC of 0.719, with explanations deemed intuitive by radiologists, showing the potential of the model in becoming a valuable tool for medical image analysis.

Recent models have been derived from ProtoPNet and applied to medical imaging, such as InterNRL [33] and IAIA-BL [3]. InterNRL, a teacher-student model, leverages ProtoPNet to identify class features and predict class probabilities, showing promising results on mammogram, retinal disease, and brain tumor datasets. IAIA-BL classifies breast masses in mammograms, utilizing detailed clinical annotations for improved accuracy and interpretability. Both models demonstrate performance on par with or superior to traditional black-box models, highlighting the potential of XAI in healthcare.

To the best of our knowledge, there are no existing studies where ProtoPNet has been used for DBT scans, and applications to the classification of breast lesions in DBT scans are not documented yet.

## 2 Methods

In this section, we delineate the comprehensive methodologies employed in our study. Initially, we elucidate the selection and preparation steps of the dataset, detailing the criteria for inclusion and the preprocessing techniques applied to ensure data integrity and relevance. Subsequently, we provide a concise overview of the architectures utilized within our research, specifically YOLOv5, YOLOv8, and ProtoPNet.

Further, we describe the training and evaluation process for both the detection and classification modules. This includes the configuration of hyperparameters, the optimization strategies adopted, and the metrics used to assess model performance.

Lastly, we highlight the clinical feedback mechanism implemented in our study. Two experienced radiologists were engaged to provide their expert analysis on the outcomes of both the detection and classification modules. Their insights were invaluable in better understanding our model and comparing the results with their clinical expectations.

### 2.1 Dataset selection and preparation

As far as we are aware, to this day, only one publicly available DBT dataset exists, i.e., the BCS-DBT dataset [5], hosted on The Cancer Imaging Archive.^2^ The dataset comprises 22032 reconstructed DBT volumes, from 5060 patients in 5610 studies, and already provides the split into the training, the validation, and the test sets. Of those, however, only the images from the training set come equipped with the corresponding malignancy label and could, therefore, be used in our analysis. It is composed of 19148 DBT scans, provided as DICOM files, from 4362 patients in 4838 studies. Items from this set are labeled as either:

• normal: no abnormal findings are present and no further imaging or pathology examinations were required;
• actionable: further imaging examination was recommended, based on a mass or architectural distortion found in the study report, however, no biopsy was performed as a result;
• benign: a mass, or an architectural distortion, present in the study underwent a biopsy, which resulted in a benign outcome;
• cancer: a mass, or an architectural distortion, present in the study underwent a biopsy, which resulted in a cancerous outcome.

For the biopsy-proven cases, i.e., those labeled as either benign or cancer, the annotations of the lesions, determined by one of two radiologists, are provided as the coordinates of a rectangular bounding-box that contains them in their central slice.

Since in our work, we are interested in the classification of benign vs. cancerous lesions, we are limited to the use of the last two classes, a fortiori, since those are the only classes of images providing the ground-truth bounding-boxes for the lesions, which are crucial for the detection step of our study. This dramatically reduces the number of cases that we can use, ending up with just 200 DBT scans from 101 patients. The distinction of the data in the two classes is reported in Table 1.

**Table 1:** Distribution of DBT scans and patients across the benign and cancer lesion classes used in this study.

Label	Scans	Patients
Benign	124	62
Cancer	76	39

**Fig. 3:**
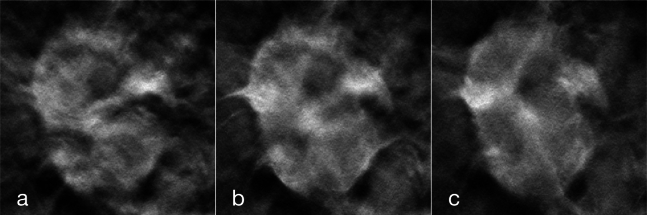
Example of some of the slices used as inter-slice data augmentation on a lesion. For visibility purposes, we show its effect on lesion crops. Different slices of the same lesion are used to enhance the variety of the dataset.
• *standard data augmentation*: this augmentation includes standard geometric transformations, applied, in turn, to each image, such as translations, rotations, and image-flipping, as well as some gray-level transformations for the classification module. A detailed description of the transformations applied, for each of the two steps of our framework, is reported in Table 2. For the detection step, we utilized the online functions for data augmentation of the YOLO library. For the classification step, we performed offline data augmentation using the torchvision.transforms module (for shift and rotation) and the Augmentor library (for skew, shear, and the two flips). Fig. 4 reports some examples of offline augmentations, applied to a lesion crop, employed for the classification module.

To, at least partially, overcome the extremely limited size of the dataset, we considered data augmentation as a pivotal step of our work. We utilized two types of data augmentation:

• *inter-slice data augmentation*: although our approach employs 2D images, to take advantage of the volumetric nature of DBT acquisitions, we exploited multiple slices of a lesion, assigning the same label to each of them. A similar approach has been used only in few other papers, such as [4] and [35]. Given the unbalance of the two classes, we selected 7 slices for benign cases and 9 slices for cancerous cases to partially balance the dataset. When possible, non-contiguous slices were selected. Since the supplied *csv* file provides just the central slice of each annotated lesion, we determined the slices in which a lesion is present, according to the guidelines,^3^ where it is stated that a lesion spans for 25% of the volume slices, in each direction, starting from the annotated slice. The benefit of this augmentation technique is that, rather than merely increasing the number of training samples through standard data augmentation methods in medical imaging, this procedure enhances the dataset by leveraging the connection between sequential slices. An example of its effect on a lesion, is reported in Fig. 3. This approach not only augments the quantity of training data but also substantially enriches the quality and diversity of the information within the samples;

**Table 2:** Data augmentation transformations used in the two steps of our framework. Since in the classification step, we utilized off-line data augmentation, we also report for that the number of random draws (N. D.) in the range of values for the parameter.

Step	Transformation	Range	N. D.
Detection	Shift	(−0.1,0.1)	
	Scale	(−0.5,0.5)	
	Flip Left-Right	Probability =0.5	
	Flip Up-Down	Probability =0.5	
	Mosaic	0	

Classification	Shift	(−0.1,0.1)	2
	Rotation	(−170,170)	10
	Skew	Magnitude =0.2	10
	Shear	(−10,10)	10
	Flip Up-Down		1
	Flip Left-Right		1
	Histogram eq.		1
	Brightness	(0.8,1.2)	1
	Contrast	(0.6,1.5)	1

**Fig. 4:**
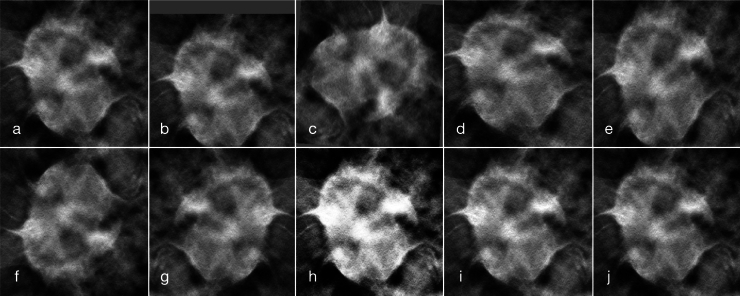
Example of some offline standard data augmentation transformations applied to a lesion crop for the classification step. Represented are the original image (a), applied shift (b), applied rotation (c), applied skew (d), applied shear (e), applied flip up-down (f), applied shift left-right (g), applied histogram equalization (h), applied brightness modification (i), and applied contrast modification (j).

**Fig. 5:**
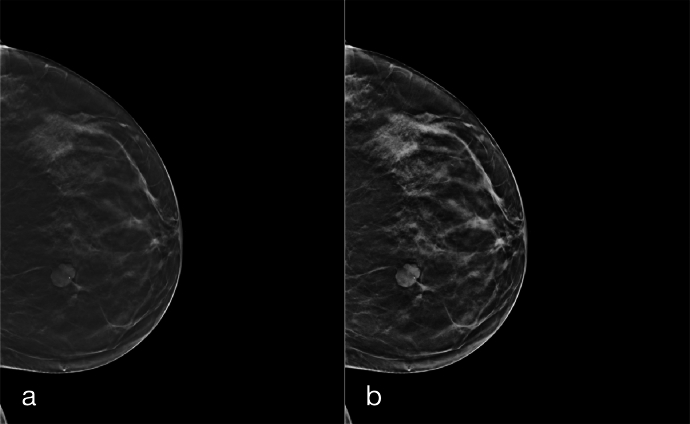
Example of the application of CLAHE to a DBT slice, a preprocessing step applied to images for the classification module. In (a) is the original image, and in (b) is the image with CLAHE.

According to the inter-slice data augmentation process, we extrapolated the selected slices from the DICOM file of the DBT scans and converted them into PNG images. For the detection step, we resized them to 1280×1050 and then applied contrast-limited adaptive histogram equalization (CLAHE) [20] to each entire slice and utilized them without any cropping. An example of the application of CLAHE to the entire DBT slice is reported in Fig. 5. For the classification step, on the other hand, we cropped the lesion with the minimal square box containing the ground-truth bounding box and then resized it to 224×224. For each patient, we utilized the same slices for the two steps. We, then, performed a patient-based partition of the dataset using an 80-20 split to separate the test set from the initial data. Subsequently, the remaining 80% was further divided using another 80-20 split to create the training and validation sets. This resulted in the dataset being allocated as follows: 64% for training, 16% for validation, and 20% for testing.

### 2.2 YOLO

The first version of YOLO was published in 2016 at the Computer Vision and Pattern Recognition Conference (CVPR) in [21] and has evolved through various iterations in the following years. The architecture is structured into three main components:

• *Backbone*: extracts features from input images using a CNN, typically pre-trained on datasets like ImageNet. It captures a hierarchy of features, from basic edges and textures to complex object parts and semantic content.
• *Neck*: enhances the features extracted by the backbone, focusing on spatial and semantic details across scales, often employing additional convolutional layers or feature pyramid networks.
• *Head*: uses refined features to predict outcomes, with subnetworks for classification, localization, and in newer versions, instance segmentation and pose estimation. Post-processing, such as non-maximum suppression, refines the predictions.

Among the various iterations of YOLO, in our work, we utilized:

• *YOLOv5*[15]: launched in 2020 by Ultralytics on the PyTorch framework, it is renowned for its speed and precision and stands at the forefront of real-time object detection technologies. Utilizing a single-stage, anchor-free methodology, it evaluates images in one forward pass, directly delineating object boundaries as well as predicting their class. Enhancements from earlier YOLO versions comprise cross-stage partial connections to retain high-level semantic information and an innovative anchor-free detection system.
• *YOLOv8*[14]: released in 2023 by Ultralytics, it is designed to improve both speed and precision in object detection. It incorporates the CSPDarknet53 as its new backbone network and introduces a novel anchor-free detection head. The system generates bounding box predictions on a per-pixel basis, while the integration of feature pyramid networks facilitates the identification of objects across a range of sizes.

### 2.3 ProtoPNet

The central idea behind ProtoPNet is to mimic the way humans approach challenging classification tasks. When we look at an image, we often dissect it and identify prototypical features that are characteristic of a particular class. ProtoPNet employs a similar strategy by dissecting images into prototypical parts and using these parts as evidence for making a final classification decision.

**Fig. 6:**
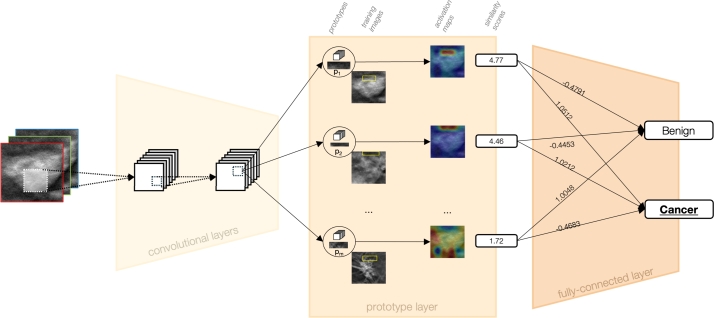
Representation of ProtoPNet Architecture. Initially, convolutional layers extract semantically meaningful features from the input image. Subsequently, a prototype layer evaluates these features against class-specific prototypes, i.e., distinctive patterns learned during training via clustering algorithms. Each comparison yields a similarity score. Finally, a fully-connected layer integrates these scores to predict the class with the highest cumulative similarity, thus determining the image's classification.

Fig. 6 shows a representation of the architecture of ProtoPNet. At its core, ProtoPNet is built upon a CNN model, such as VGGs, ResNets, and DenseNets, which is used for initial feature extraction. This convolutional block is then followed by a *prototype layer* and a *fully connected layer*. The prototype layer is particularly crucial as it contains a predetermined number of class-specific prototypes, learned at training time.

The prototype layer distinguishes ProtoPNet from conventional CNNs by learning prototypical parts, i.e., the *prototypes*, of the classes during training. These prototypes are the centroids derived from a clustering algorithm applied to patches of the training images. Consequently, the training phase loss function is a weighted sum of the cross-entropy and two additional terms: the *clustering* term, which ensures each training image has a latent patch close to at least one prototype of the same class, and the *separation* term, which keeps the training patches distant from prototypes of different classes.

The prototypes are, therefore, learned such that each one represents a part that is crucial for the identification of a class (e.g., the beaks, wings, paws, etc., of different species in a bird classification task). In the case of the classification of benign and cancer breast masses, those prototypes could be something like, for instance, the smooth or rough edges of masses, a particular pattern in the breast tissue, and so on. During the inference phase, the network compares parts of a new image with these learned prototypes to find similarities. The similarities are then used to generate an *activation map*, which highlights the parts of the image that most strongly match the prototypes. From the activation map, via max pooling, a *similarity score* is also obtained that quantifies the adherence of a certain patch of a test image to a prototype.

The final layer, the *fully connected layer*, combines the similarity scores to weigh the evidence from the prototypes and make a classification decision. The activation function for the first two types of layers is the Rectified Linear Unit (ReLU), while the last layer uses the sigmoid activation function. This structure allows ProtoPNet to not only identify the class of an image but also to provide a visual explanation by showing which prototypical parts influenced the decision more.

The training of ProtoPNet is a multi-stage process that begins with stochastic gradient descent on all layers except the last. After a series of *warm-up epochs* with fixed pre-trained weights and biases, the *joint epochs* begin, where the convolutional block and the prototype layer are trained together. This allows the prototypes to be shaped by both the raw image data and the emerging classification patterns. Second, prototypes are projected onto the closest latent representation of training images' patches, which allows them to correspond to a real path of a training image. Lastly, the *optimization of the last layer* is performed, fine-tuning the decision-making process by adjusting the fully connected layer's parameters to best utilize the evidence provided by the prototypes.

### 2.4 Training process

After the dataset preparation, as described in Section 2.1, the training of our framework proceeded in two stages. Initially, the detection module was trained, followed by the training of the classification module. For both modules, we utilized internal training and validation sets to tune the hyperparameters. Once the optimal configuration for both modules was determined, we evaluated their performances on the test set.

#### 2.4.1 Training of the detection module

The training of the detection module consisted in a fine-tuning of two versions of the YOLO model, i.e., YOLOv5 and YOLOv8, on our dataset. We investigated the best configuration of hyperparameters by performing a grid-search on the learning rate, learning rate scheduler, optimizer, and weight decay. The values explored in the grid-search for each hyperparameter are reported in the Supplementary Materials.

We also explored an ensemble of YOLOv5 and YOLOv8 models to check if this could benefit the overall performance on the dataset. For this purpose, we combined the predictions obtained with the best-performing YOLOv5 and YOLOv8 models on the same images. When comparing the predictions for an image, two predictions were considered identical if their intersection over union (IoU), defined as:

$$IoU=\frac{\text{area of intersection}}{\text{area of union}}$$

was greater than a threshold of 0.3. In case of a positive match, the confidence level, which represents the model's certainty that a detected object is indeed a lesion and that the bounding box accurately encloses it, of the two predictions were combined by performing the mean of the confidence of each model on that prediction, to obtain the confidence of the ensemble. Therefore, the resulting confidence of a combined prediction is given by:

$$conf_{\text{ensemble}}=\frac{conf_{\text{v5}}+conf_{\text{v8}}}{2}$$

### 2.5 Evaluation of the detection module

Based on the performance on the internal validation set, we chose the number of epochs with which we re-trained the model on the whole training set. We then tested it on the test set. We evaluated the models of the detection module based on the metrics of *Recall* (*R*) and *Precision* (*P*), defined as follows:

$$R=\frac{TP}{TP+FN}$$

$$P=\frac{TP}{TP+FP}$$

Where *TP* is the number of true positive findings, *FN* the number of false negative findings, and *FP* the number of false positive findings.

For the YOLOv5 and YOLOv8 models, we evaluated the predictions by considering as relevant only those with an IoU with the ground-truth annotation greater than 0.1 and a confidence value greater than 0.1. For the ensemble model, we set the threshold on the confidence value to 0.05, to render it comparable to the single models.

#### 2.5.1 Training of the classification module

For the classification module, we utilized ProtoPNet with ResNet18 convolutional layers as the convolutional block. We detail the grid-search process for optimizing the values of the hyperparameters in the Supplementary Materials.

After the training process, we also performed the pruning process, as described in [7]. This step aims at removing the prototypes that are less important or redundant from the model.

To compare our results with those of a standard black-box model, we also followed similar training steps to train a ResNet18 on the same dataset. To the plain version of ResNet18, we added a 2D dropout of 0.2 on the last convolutional layer and a dropout of 0.4 on the final fully connected layer. In that configuration, the grid search was used to optimize the learning rate, weight decay, and the parameters of the learning rate scheduler.

### 2.6 Evaluation of the classification module

We conducted an evaluation on the test set. In addition to Precision and Recall, defined as in 2.5, and to auROC, we assessed the performance of the model by referring to the metrics of Balanced Accuracy (B. Acc.) and Specificity (*S*) defined as:

$$\text{B. Acc.}=\frac{1}{2}\left(\frac{TP}{TP+FN}+\frac{TN}{TN+FP}\right)$$

$$S=\frac{TN}{TN+FP}$$

### 2.7 Clinical feedback

As part of our commitment to transparency and clinical relevance, we sought the expertise of two experienced radiologists to evaluate the performance of our AI system both in detecting lesions in DBT scans and in classifying them in an explainable fashion. Their feedback was instrumental in assessing the practicality of our framework and its potential integration into clinical workflows.

#### 2.7.1 Feedback on the detection module

The first aspect of the evaluation involved a comparison between the detection results of our AI system on the test set and the provided ground truth bounding boxes. The radiologists were tasked with verifying the accuracy of these ground truth annotations. This step was crucial, as it served as the benchmark for our system's performance. The radiologists scrutinized the bounding boxes of the test-set images for consistency and relevance, identifying any discrepancies that could impact the detection accuracy.

The second aspect of the feedback process addressed the clinical significance of the false positives identified by our AI system. It is not uncommon for AI detection systems to flag non-lesion areas as potential concerns. Therefore, we asked the radiologists whether these false positives represented clinically meaningful findings or were merely normal breast tissue (parenchyma).

#### 2.7.2 Feedback on the classification module

The classification module of our AI framework, based on ProtoPNet, introduces a novel approach to medical image analysis by utilizing self-learned prototypes for classification. The inherent complexity of applying ProtoPNet to medical images necessitates a deep understanding of the clinical context to fully interpret its explanations. Therefore, we asked the two radiologists to provide their feedback on three critical aspects of the classification module.

The first aspect concerned the clinical significance of the learned prototypes. The radiologists were asked to assess whether these prototypes were clinically relevant markers for distinguishing between benign and malignant lesions, by assigning to each prototype a relevance score from 1 to 5.

The second aspect regarded the evaluation of the soundness of the concept of similarity learned by ProtoPNet. This involved a comparison between the mathematically-based concept of similarity, as utilized by ProtoPNet, and the radiologists' concept of similarity, which is grounded in their extensive on-field experience. For this purpose, they were presented with 22 random similarities between images and prototypes used by the model during inference, and they were asked to rate their agreement with the identified similarity on a scale from 1 to 5.

Lastly, the radiologists assessed their agreement with the explanations provided by ProtoPNet. They were presented with 22 random lesions classified by the model, along with explanations showcasing the 5 most highly activated prototypes. Radiologists scored their trust in the classification, on a scale from 1 to 5, based on these explanations. Given the pivotal role of ProtoPNet's explanations in its interpretability and trustworthiness, this feedback was indispensable.

## 3 Results

In this section, we report the results obtained from the training and evaluation of the two modules of our pipeline. We also disclose what was obtained from the clinical feedback for both modules, which was instrumental in gaining a deeper understanding of the training results.

### 3.1 Results for the detection module

The inter-slice data augmentation and the split process were instrumental to the training phase. The detailed numbers resulting from this process are presented in Table 3, which pertains to the dataset used for the detection module.

**Table 3:** Detection step: after the inter-slice data augmentation, the number of images considered in the training set, in the validation set, as well as in the test set.

Set	Images
Training	937
Valid	226
Test	296

The resulting performance metrics are reported in Table 4, those were obtained with the hyperparameters reported in the Supplementary Materials, as a result of the grid-search process. In the same table, we have also included the metrics for the ensemble method, obtained as described in Section 2.4.1. Among the three approaches, the ensemble method exhibits the highest recall, indicating the greatest number of true positive detections, albeit with the lowest precision, leading to the highest number of false positives. On the other hand, YOLOv5 has the second-best recall, with a precision comparable to that of the ensemble. Conversely, YOLOv8 provides the best compromise between precision and recall. A more detailed analysis of the false positives identified by the models, in light of clinical feedback, will be presented in Section 3.3.1.

**Table 4:** Evaluation metrics resulting for the two YOLO models, as well as for the ensemble method, on the test set.

Model	Recall	Precision
YOLOv5	0.68	0.32
YOLOv8	0.62	0.61
Ensemble	0.76	0.31

### 3.2 Results of the classification module

As described in Section 2.1, for the classification module, we applied both inter-slice data augmentation and off-line standard data augmentation. The application of the split process and the inter-slice data augmentation resulted in the number of images reported in Table 5. Subsequently, applying off-line standard data augmentation to these images yielded the numbers presented in Table 6.

**Table 5:** Classification step: number of images for each class after inter-slice data augmentation, divided into the training, validation, and test sets.

Set	Benign Images	Cancer Images
Training	595	486
Valid	154	108
Test	196	162

**Table 6:** Classification step: final number of images, for each set, after the application of both off-line standard data augmentation as well as inter-slice data augmentation.

Set	Benign Images	Cancer Images
Training	22610	18468
Valid	5852	4104
Test	7448	6156

**Fig. 7:**
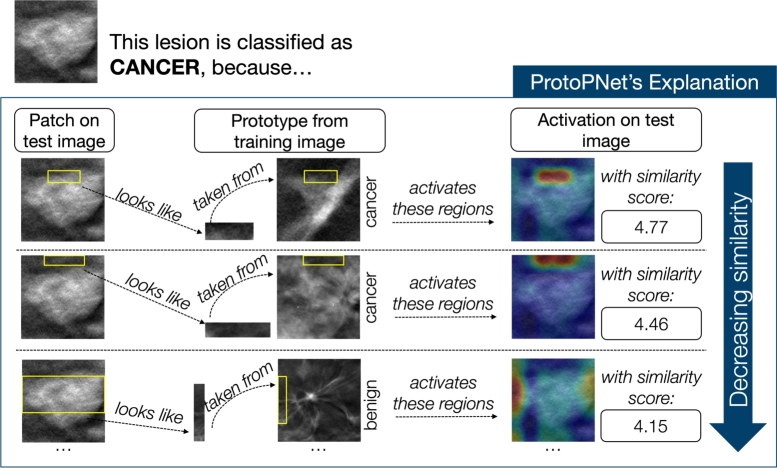
This figure illustrates how ProtoPNet successfully identifies a lesion as malignant. It does so by comparing patches of the image with the learned prototypes. Displayed within the figure are the top three prototypes that most closely match the lesion, arranged in order of their similarity, from highest to lowest.

The best performing configuration gave, on the validation set, a Balanced Accuracy of 0.77, a Precision of 0.71, a Recall of 0.76, a Specificity of 0.79, and an auROC of 0.81. We report, in the Supplementary Materials, the hyperparameters that lead to those results. When tested on the never-before-seen test set, we obtained the metrics reported in Table 7. Fig. 7 reports an example of the explanation provided by ProtoPNet for a lesion that is correctly classified as cancerous. This is based on the similarities between image patches and the learned prototypes. In the figure, we present the first three most strongly activated prototypes in decreasing order of similarity. As a point of comparison, we evaluated ResNet18, using the hyperparameters resulting from the grid-search, reported also in the Supplementary Materials. This configuration achieved, on the test set, the metrics reported in Table 8.

**Table 7:** Evaluation metrics on the test set resulting for the best-performing ProtoPNet configuration.

Metric	Value
B. Accuracy	0.70
Precision	0.70
Recall	0.67
Specificity	0.74
auROC	0.66

**Table 8:** Evaluation metrics on the test set resulting for the best-performing baseline ResNet configuration.

Metric	Value
B. Accuracy	0.64
Precision	0.64
Recall	0.58
Specificity	0.72
auROC	0.64

### 3.3 Results of the clinical feedback

In the following two subsections, the results from the clinical feedback are presented. Subsection 3.3.1 collects the observations on the detection module results and Subsection 3.4 the ones from on the classification module.

#### 3.3.1 Feedback on the detection module

From the clinical feedback provided by the two radiologists concerning the performance of the detection module, two significant observations were made:

1. *Ground Truth Annotation Accuracy*: the ground truth annotation, guided by official guidelines,^4^ which state that a lesion should span for 25% of the volume slices in each direction starting from the annotated slice, is not always precise. This imprecision is particularly evident in scans with multiple lesions, where a lesion may be visible in a slice annotated for a different lesion, yet the bounding box for the visible lesion is absent. In Fig. 8, we present an example of a lesion which, according to the guidelines, should not be present in a slice. Despite this, it was visible and our model identified it, indicating a potential error in the ground truth annotations.

**Fig. 8:**
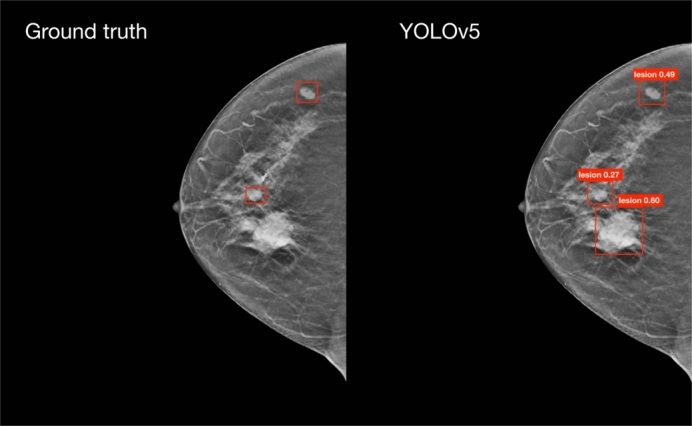
On the left, the image displays the ground truth bounding boxes, while on the right, the bounding boxes predicted by YOLOv5 are shown. This example highlights a lesion (the one at the bottom) that, according to the official guidelines, should not be visible in this slice, since they state that a lesion spans 25% of the volume slices in each direction from the annotated slice. Contrary to the guidelines, the lesion is visible, and YOLOv5 accurately identifies it, indicating an error in the ground truth annotation for this particular slice.
2. *Classification of False Positives*: a considerable number of detections classified as false positives were not normal breast tissue. Instead, they were clinical entities such as, among others, non-annotated lesions, lymph nodes, glandular thickening, cysts, and masses with low positive predictive value (PPV), that is, the probability that a test is actually positive. Fig. 9 shows an example of a lesion with low PPV that was identified by our model, but was not present in the ground truth annotations.

**Fig. 9:**
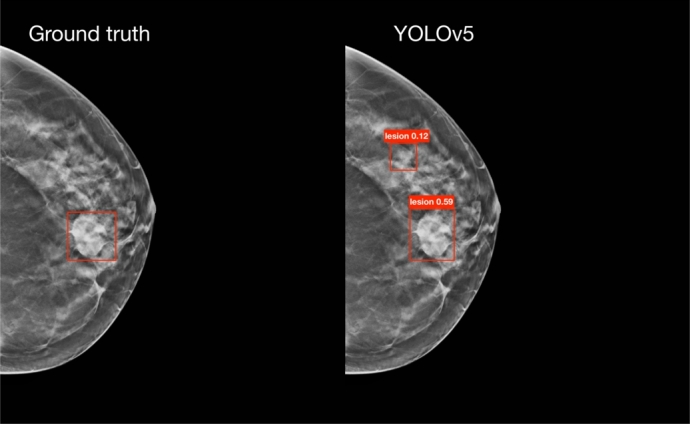
On the left, the image displays the ground truth bounding boxes, while on the right, the bounding boxes predicted by YOLOv5 are shown. This example highlights a lesion (the one at the top) with low positive predictive power (PPV) that was not present in the ground truth annotations, but was identified by one of our models.

Regarding the false positives identified by the model, one radiologist observed that the model tends to make more errors, specifically predicting a higher number of false positives, when analyzing highly dense breasts. These are challenging cases where even experienced radiologists would flag a greater number of ambiguous cases for further examination by ultrasound. She also stated:

*The model makes errors not too dissimilar from those a radiologist might make; it errs as a radiologist could. It depends a lot on experience, as they too can make mistakes and confuse, for example, a glandular island with a mass, thus requiring further investigation with ultrasound, especially in very dense breasts. However, some of these uncertain cases are already excluded by comparing multiple consecutive slices.*

As a result of this observation, a potential future improvement could involve combining adjacent slices into the three channels of an RGB image. This approach may help reduce the number of false positives in challenging cases and enhance the overall performance of the model.

To adopt a safer approach, we could consider submitting all clinical entities identified by the detection module for further assessment by a physician. By categorizing these entities as true positive findings, the evaluation metrics would align with those presented in Table 9. These metrics show a visible improvement compared to those in Table 4. Such reclassification could enhance the clinical utility of the detection module by providing a more comprehensive overview of detectable anomalies.

**Table 9:** Evaluation metrics for the two YOLO models, as well as for the ensemble method on the test set, after incorporating the corrections from the clinical feedback.

Model	Recall	Precision
YOLOv5	0.79	0.56
YOLOv8	0.67	0.66
Ensemble	0.80	0.58

### 3.4 Feedback on the classification module

Analysis of feedback from two radiologists on the ProtoPNet classification module yielded three key insights: the clinical significance of the learned prototypes, the adherence to concepts of similarity between ProtoPNet and radiologists, and the overall trust in the classifications, based on the explanations.

**Fig. 10:**
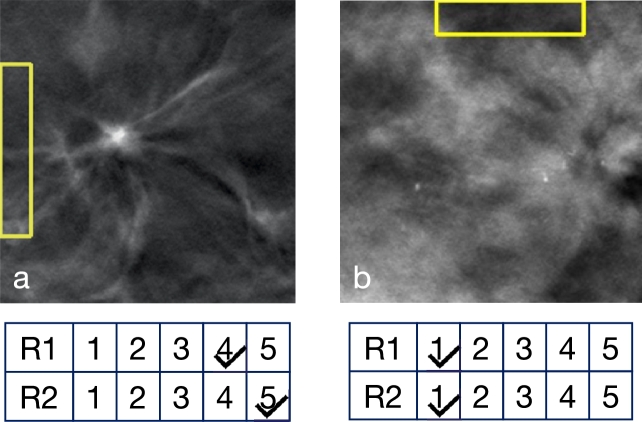
Two examples of scores obtained from clinical feedback on the significance of prototypes. The training images from which the prototype (in the yellow box) was selected are shown. Below each image are the scores assigned by Radiologist 1 (R1) and Radiologist 2 (R2). (a) Example of a prototype receiving a high score. (b) Example of a prototype receiving a low score.

*Clinical significance of prototypes*  The clinical significance of benign and malignant prototypes was evaluated by the radiologists, who assigned scores to five prototypes from each class. The distribution of scores and average sentiment scores are summarized in Table 10. In general, the benign prototypes were deemed to have more acceptable clinical significance compared to the malignant ones. In Fig. 10, we report two examples of scores assigned to prototypes: in Fig. 10a an example of a (benign) prototype that received a high score, in Fig. 10b an example of a (malignant) prototype that received a low score.

**Table 10:** Radiologists' assessment of the clinical significance of benign and malignant prototypes (scores ≥3 indicate acceptable significance).

Prototype	Radiologist 1	Radiologist 2	Average Sentiment Score
Class	(Scores ≥3)	(Scores ≥3)	(R1/R2/Overall)
Benign	3 out of 5	4 out of 5	3.2 / 3.6 / 3.4
Malignant	2 out of 5	0 out of 5	2.2 / 1.6 / 1.9

**Fig. 11:**
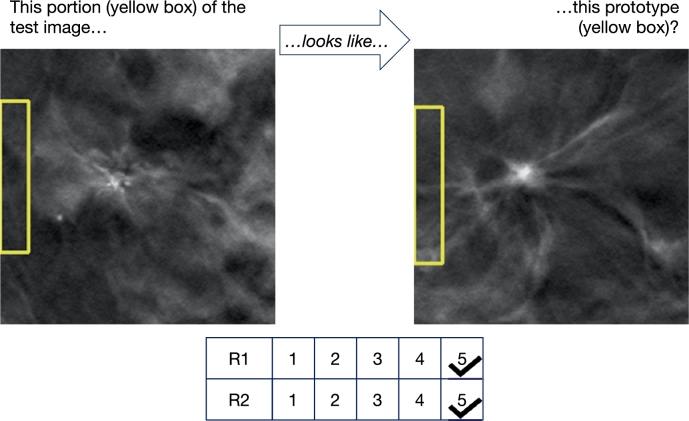
Example of a similarity utilized by ProtoPNet during classification, which received high scores from both Radiologist 1 (R1) and Radiologist 2 (R2), indicating that it adheres to their concept of similarity.

**Fig. 12:**
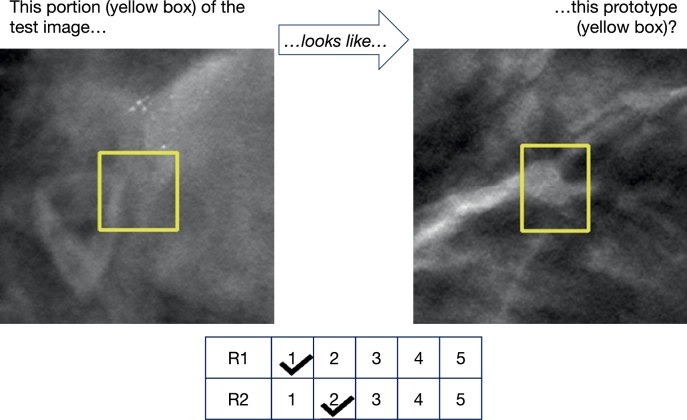
Example of a similarity utilized by ProtoPNet during classification, which received low scores from both Radiologist 1 (R1) and Radiologist 2 (R2), indicating that it does not adhere to their concept of similarity.

*Adherence in concepts of similarity*  The adherence of ProtoPNet's concept of similarity to that of the radiologists was assessed on 22 randomly-selected cases. Table 11 summarizes the number of cases receiving a score of at least three (indicating adherence) and the average sentiment scores. Examples of high-scoring and low-scoring similarity cases are shown in Fig. 11 and Fig. 12, respectively. The combined average sentiment score suggests a broadly neutral stance on the alignment of similarity concepts.

**Table 11:** Radiologists' assessment of the adherence of ProtoPNet's similarity concept (scores ≥3 indicate adherence).

Prototype	Radiologist 1	Radiologist 2	Average Sentiment Score
Class	(Scores ≥3)	(Scores ≥3)	(R1/R2/Overall)
Similarity Adherence	14 out of 22	10 out of 22	3.1 / 2.6 / 2.85

*Trust in the classifications*  The radiologists' trust in the classifications based on ProtoPNet's explanations was evaluated on the same 22 cases. Table 12 presents the number of cases with a score of at least three (indicating trust) and the average sentiment scores. The results indicate a slightly negative overall trust in the classifications based on the provided explanations.

**Table 12:** Radiologists' trust in the classifications based on ProtoPNet's explanations (scores ≥3 indicate trust).

Prototype	Radiologist 1	Radiologist 2	Average Sentiment Score
Class	(Scores ≥3)	(Scores ≥3)	(R1/R2/Overall)
Trust in Classification	13 out of 22	7 out of 22	2.9 / 2.2 / 2.55

As a conclusion of the investigation, one of the radiologists also stated:

*There are aspects in the AI's explanation of the judgment that, in my opinion, are not consistent with the semiotics [author's note: the study of signs and symbols and their use or interpretation, here, specifically, from medical images, which lead to the diagnostic decision] as we have studied it as radiologists. However, the computer probably reads in a different grayscale than the semiotics we are used to.*

The radiologist, therefore, observed a misalignment between the explanations from the network and the traditional methods and signs that radiologists are trained to recognize and interpret on the images. However, she suggests that the model might be analyzing the images using a different range of grayscale values than what radiologists typically use. This could lead to different interpretations and conclusions.

## 4 Discussion

This study introduced a novel AI system designed for transparent lesion analysis in 2D DBT slices, capable of both localization and classification while providing an interpretable rationale. Our two-stage approach leverages deep learning for lesion detection via state-of-the-art neural networks, specifically YOLOv5 and YOLOv8 (including an ensemble strategy), followed by ProtoPNet for benign/cancerous classification based on prototypical part learning, thus enhancing the interpretability of the results.

While the performance of our method demonstrates promise, further refinement is needed before seamless integration into clinical workflows. Comparing our detection module to the DBTex challenge [16], the top-performing model achieved a recall of 0.92. Our ensemble approach yielded a recall of 0.76, which improved to 0.80 after incorporating expert radiologist feedback that addressed ground-truth annotation inaccuracies. Although lagging behind the winning entry, our performance is competitive with other challenge participants who, like us, solely utilized the publicly available BCS-DBT dataset.

Direct comparison of our classification module is challenging due to the limited research specifically focused on classifying lesions in DBT images. While some studies address the simpler task of distinguishing cancerous lesions from normal tissue, [37] remains a key work in benign/malignant mass classification. Despite their superior performance, the use of a private dataset makes direct benchmarking less relevant.

A significant hurdle in this research was the limitation of the BCS-DBT dataset, the only publicly accessible collection of DBT images. A substantial portion of the dataset lacked lesions, rendering them unusable. The resulting small number of relevant cases necessitated the use of standard and inter-slice data augmentation to artificially expand our training set by treating multiple slices of a single lesion as independent instances. However, we acknowledge that such augmentation cannot fully replicate the diversity inherent in a large, varied dataset.

In spite of these challenges, this work provides a crucial proof of concept for applying explainable-by-design AI to DBT image analysis for lesion characterization. Given the scarcity of alternative public datasets, our initial results, though requiring further development, represent a valuable starting point with significant potential for future improvement. We are actively addressing these limitations through dedicated efforts to refine the existing public dataset and the ongoing collection of a private dataset within the “Mortalità zero” project, of the Fondazione Umberto Veronesi, which we anticipate will significantly increase the quantity and diversity of our training data, ultimately leading to substantial performance improvements in our presented model.

Clinical feedback on the detection module highlighted further dataset issues. Radiologists identified frequent *inaccuracies in ground truth annotations*, particularly in multi-lesion scans where bounding boxes were often missing. This suggests potential errors within the training data itself. Moreover, many false positives identified by our model were recognized as clinically relevant entities by radiologists, such as unannotated lesions, lymph nodes, or masses with low Positive Predictive Value.

The dataset's categorization of lesions into masses and architectural distortions also presented a challenge. Analyzing these categories separately would have severely reduced the already limited number of cases per class, necessitating their aggregation despite the increased complexity this introduced to the classification task.

These data limitations also impacted the clinical evaluation of our classification module. While benign prototypes were generally considered clinically meaningful, cancerous prototypes received a negative assessment. Furthermore, the agreement between the mathematical concept of similarity used by ProtoPNet and the clinical intuition of radiologists was neutral. Overall trust in the classifications, based on the provided explanations, was slightly negative. One radiologist suggested this might stem from the model analyzing a different range of grayscale values than those typically used by clinicians for identifying significant features. Addressing these dataset limitations with a larger and more diverse collection is likely to improve these aspects significantly.

Our work presents several key innovations. Firstly, we developed a comprehensive, end-to-end interpretable framework for breast lesion identification and classification, offering significant potential for medical applications contingent on the availability of larger, higher-quality datasets. This framework provides not only a classification (benign or cancerous) but also an explanation for that decision. Secondly, the application of ProtoPNet to the complex medical challenge of DBT analysis is novel. This is significant as ProtoPNet offers an inherently interpretable decision-making process, crucial in medical diagnostics. Finally, the application domain itself is cutting-edge, as DBT is an evolving imaging modality, and the integration of AI in this area is still in its early stages, positioning our approach as an important contribution.

Future work should prioritize meticulous verification of the dataset's ground truth by expert radiologists to ensure accurate lesion labeling, a critical step for reliable model training and evaluation. Simultaneously, expanding the dataset through the incorporation of new public and private data sources is crucial for improving the model's generalizability and robustness. Building upon clinical feedback, we will explore combining adjacent DBT slices into RGB channels as a potential method to reduce false positives in the detection module, with a longer-term goal of transitioning to a full 3D implementation to leverage the inherent volumetric information of DBT scans. To further enhance performance, future efforts will also focus on rigorous model optimization, including exploring advanced data augmentation techniques beyond those currently employed, refining training strategies, and potentially investigating alternative network architectures or learning paradigms. Finally, comprehensive hyperparameter optimization and cross-validation, while computationally intensive, are essential for refining model performance and ensuring robustness across diverse clinical scenarios and datasets.

## 5 Conclusion

In conclusion, our study demonstrates the potential of advanced NN models and prototypical-part networks in the analysis of DBT scans. The encouraging performance level achieved by our method, which is expected to improve substantially with the availability of more comprehensive datasets, along with the inherent interpretability of its classification, is a promising step towards the development of AI systems that can support radiologists in the detection and classification of breast cancer. In data-driven approaches like ours, increasing both the size and the quality (in terms of diversity, annotation accuracy, and reduced bias) of the training data is paramount, as it directly translates to more robust, generalizable, and ultimately more reliable AI models. Future work should aim to address the dataset limitations and explore the integration of our method into clinical workflows, ensuring that the system's transparency and interpretability remain at the forefront of its design.

## CRediT authorship contribution statement

**Andrea Berti:** Writing – original draft, Software, Methodology, Conceptualization. **Camilla Scapicchio:** Writing – original draft, Methodology, Conceptualization. **Chiara Iacconi:** Validation. **Charlotte Marguerite Lucille Trombadori:** Validation. **Maria Evelina Fantacci:** Supervision, Conceptualization. **Alessandra Retico:** Supervision, Conceptualization. **Sara Colantonio:** Supervision, Conceptualization.

## Declaration of Competing Interest

The authors declare that they have no known competing financial interests or personal relationships that could have appeared to influence the work reported in this paper.

## Appendix A Supplementary material

MMC Detailed Methodology for Hyperparameter Optimization: This section outlines the specific steps and considerations undertaken during the optimization of the model's hyperparameters. MMC

## Footnotes

3 https://sites.duke.edu/mazurowski/resources/digital-breast-tomosynthesis-database/.

4 https://sites.duke.edu/mazurowski/resources/digital-breast-tomosynthesis-database/.

## Footnotes

2 https://www.cancerimagingarchive.net/collection/breast-cancer-screening-dbt/.
