# Supplementary material for: An explainable-by-design end-to-end AI framework based on prototypical part learning for lesion detection and classification in Digital Breast Tomosynthesis images
Source: Comput Struct Biotechnol J. 2025 Jun 10;27:2649–60. doi: 10.1016/j.csbj.2025.06.008 (PMC12212108; doi:10.1016/j.csbj.2025.06.008)
Supplement: MMC — Detailed Methodology for Hyperparameter Optimization: This section outlines the specific steps and considerations undertaken during the optimization of the model's hyperparameters. [file mmc1.pdf]

Table 1
Values of the hyperparameters explored in the grid search for the optimization of the detection step in both YOLOv5 and YOLOv8. We optimized the model version, the initial learning rate (LR), the final LR fraction (LRF), the LR scheduler, the weight decay (WD), and the optimizer. Each parameter value listed in the table was systematically paired with every value of the other parameters to explore all possible configuration permutations.

Hyperparameter	Values			
Model version	Nano	Small	Medium	Large
Initial LR	$1e-6$	$1e-5$	$1e-4$	$1e-3$
LRF	$1e-4$	$1e-3$	-	-
LR scheduler	None	Linear	Cosine	-
WD	$5e-4$	$5e-3$	$5e-2$	$5e-1$
Optimizer	SGD	Adam	AdamW	Adamax

Table 2
Hyperparameter values explored in the first-step grid-search for classification optimization. This step involved optimizing the two learning rates for the warm-up epochs for the add-on layer (w_opt_lrs (add)) and the prototype layer (w_opt_lrs (proto)); the three learning rates for the joint epochs for the convolutional block (j_opt_lrs (feat)), the add-on layer (j_opt_lrs (add)), and the prototype layer (j_opt_lrs (proto)); the learning rate for the last layer (l_layer_opt_lr); and the weight decay (WD). Each parameter value listed in the table was systematically paired with every value of the other parameters to explore all possible configuration permutations.

Hyperparameter	Values			
w_opt_lrs (add)	$1e-6$	$5e-6$	$1e-5$	$5e-5$
w_opt_lrs (proto)	$1e-6$	$5e-6$	$5e-5$	-
j_opt_lrs (feat)	$1e-6$	$5e-5$	-	-
j_opt_lrs (add)	$5e-7$	$1e-6$	$5e-6$	-
j_opt_lrs (proto)	$5e-7$	$1e-6$	$5e-6$	-
l_layer_opt_lr	$1e-6$	$1e-5$	$1e-4$	-
WD	0.01	0.02	0.05	0.1

Table 3
Hyperparameter values for the second-step grid-search in classification optimization. Each parameter value listed in the table was systematically paired with every value of the other parameters to explore all possible configuration permutations.

Hyperparameter	Values		
n. prototypes	3	5	10
cluster	0.7	0.8	0.9
separation	$3e-2$	$5e-2$	$7e-2$

1. Supplementary material

1.1. Optimization of hyperparameters: detection module

For the detection module, we explored, via a grid search, the values reported in Table 1 for the hyperparameters.

1.2. Optimization of hyperparameters: classification module

Given its complexity, the optimization of the hyperparameters of the detection module was implemented via a grid search in three steps. First, we fixed the number of prototypes per class to 5 and the weights of the cluster and separation terms in the loss function to 0.9 and 0.05, respectively. With that set-up, we varied the learning rates for the joint, warm-up, and last-layer epochs, along with the weight decay, exploring the configurations in Table 1. Subsequently, for the best configuration, with a secondary grid search, we refined the number of prototypes per class and the weights for the clustering and separation terms in ProtoPNet's loss function (Table 3). Finally, a tertiary grid search fine-tuned the gamma and step parameters of the StepLR learning rate scheduler for the best-performing setup (Table 4).

Table 4
Hyperparameter values for the third-step grid-search in classification optimization. Each parameter value listed in the table was systematically paired with every value of the other parameters to explore all possible configuration permutations.

Hyperparameter	Values	
gamma	0.1	0.5
step	2	3
	5	7
	10	15
	20	-

Table 5
Results of the hyperparameter optimization for YOLOv5 and YOLOv8. We used grid search to optimize the model version, initial learning rate (LR), final LR, LR scheduler, weight decay (WD), and optimizer.

Hyperparameter	Value in YOLOv5	Value in YOLOv8
Model version	Small	Medium
Initial LR	$1e-3$	$1e-4$
Final LR	$1e-6$	$1e-7$
LR scheduler	Linear	Cosine
WD	$5e-4$	$5e-1$
Optimizer	SGD	SGD

Table 6
ProtoPNet: best performing hyperparameter configuration resulting from the grid search steps. The optimized hyperparameters were the two learning rates for the warm-up epochs for the add-on layer (w_opt_lrs (add)) and the prototype layer (w_opt_lrs (proto)); the three learning rates for the joint epochs for the convolutional block (j_opt_lrs (feat)), the add-on layer (j_opt_lrs (add)), and the prototype layer (j_opt_lrs (proto)); the learning rate for the last layer (l_layer_opt_lr); and the weight decay (WD); the number of prototypes; the cluster and separation weights in the loss function; and the learning rate scheduler (LR scheduler).

Hyperparameter	Value
w_opt_lrs (add)	$5e-6$
w_opt_lrs (proto)	$5e-6$
j_opt_lrs (feat)	$5e-5$
j_opt_lrs (add)	$5e-7$
j_opt_lrs (proto)	$1e-6$
l_layer_opt_lr	$1e-5$
WD	0.01
n. prototypes	5
cluster	0.9
separation	0.05
LR scheduler	None

1.3. Hyperparameter optimization results: detection module

We report in table 5 the best configuration for the hyperparameters for YOLOv5 and YOLOv8, as resulting from the grid search process. For the YOLOv5 model, it was obtained after 27 epochs, and for the YOLOv8 model after 25 epochs.

1.4. Hyperparameter optimization results: classification module

As a result of the hyperparameter optimization step conducted on ProtoPNet through grid search, we identified the configuration reported in Table 6, trained for 30 epochs plus 10 last-layer optimization epochs, as the best performing one. As a result of the pruning step, no prototype was removed. Table 7, on the other hand, reports the hyperparmeters that lead to the best performance for ResNet, after training for 18 epochs.

Table 7
ResNet18: best performing hyperparameter configuration resulting from the grid search. We optimized the learning rate (LR), the weight decay (WD), and the gamma and step parameters of the LR scheduler.

**Hyperparameter**	**Values**
LR	$5e-5$
WD	0.2
gamma	0.5
step	15
